# The relationship between age and driving attitudes and behaviors among older Americans

**DOI:** 10.1186/s40621-015-0043-6

**Published:** 2015-05-21

**Authors:** Alexander J Mizenko, Brian C Tefft, Lindsay S Arnold, Jurek G Grabowski

**Affiliations:** AAA Foundation for Traffic Safety, 607 14th Street, NW, Suite 201, 20005 Washington, DC USA

**Keywords:** Traffic, Safety, Seniors, Driving, Aging

## Abstract

**Background:**

Due to a decreasing birth rate and longer life expectancy, the proportion of Americans over the age of 65 is expected to rise in coming years. Drivers over 65 drive two billion miles yearly, a number that will increase. For that reason, it is imperative to understand their attitudes and perceptions. It is also important to understand whether drivers over 65 can be treated as one cohesive group, or if there are differences among them.

**Methods:**

A web-enabled survey was conducted among Americans in the years 2011–2013. Responses from 1793 persons over 65 regarding attitudes towards driving behaviors, support for safety interventions, and engagement in unsafe behaviors were analyzed. Respondents were stratified by age: 65–69, 70–74, and 75 and older. Age groups were compared using logistic regression. Other potential explanatory factors were analyzed and controlled for.

**Results:**

The three groups were similar on many outcomes. However, statistically significant differences were found between them with regard to perceptions on speeding and the support for speed cameras, among other outcomes. In nearly all cases, those 75 and older were the most “pro-safety.” However, when adjusted for demographic characteristics other than age, a larger proportion of respondents 75 and older reported engaging in red light running and drowsy driving in the last 30 days, and the difference was statistically significant.

**Conclusion:**

Older drivers are strongly “pro-traffic safety.” However, the finding that those 65–69 are less so is concerning. This is especially true if it is the result of a cohort effect instead of an age effect. The increase in certain behaviors among those 75 and older is also concerning; drivers over this age are more prone to fatal injury when involved in a motor vehicle crashes. This poses a public health issue as the 75and older population expands.

## Background

The United States is at the edge of a major demographic shift in which the overall population characteristics will move from the middle aged workers to older retirees because people aged 65 and over make up the fastest growing population segment and this trend is predicted to continue for the next several decades. In 1900, people ages 65 years and older comprised only 4 % of the population of the United States. By 2000, their share of the population had tripled to 12 %, and is predicted to reach 20 % by 2050 (Hobbs et al. 1900; United States Census Bureau. Table 12. Projections of the Population by Age and Sex for the United States 2010). Nearly 75 % of today’s adults over 65 say they are in good to excellent health (Behavioral Risk Factor Surveillance System Survey 2012), 42 % report they have attended some college (United States Census Bureau. Table 231. Educational Attainment by Selected Characteristics 2010) and the median income is $33,848 (DeNavas-Walt et al. 2012). Older Americans are living active lives in which many still hold careers and participate in community and religious groups; some seniors are even caring for older parents (Bateson 2013).

According to research by the AAA Foundation for Traffic Safety, Understanding Older Drivers: An Examination of Medical Conditions (2014a), 86 % of Americans ages 65 and older still drive, meaning that there are presently approximately 36 million drivers ages 65 and older. If current population projections and licensure rates hold, this number will grow to 48 million by 2020. Drivers ages 65 and older drove an estimated 219 billion miles in the one-year period from May 2008 through April 2009 (Federal Highway Administration 2009).

As older people remain healthier for longer, driving will undoubtedly continue to play a major role in their ability to stay mobile, independent, and engaged in their community. Hence, there is a need to better understand and delineate the changes that occur in the attitudes and perceptions of older drivers as they continue to age. This is especially true because older adults are sometimes perceived as being an unsafe presence on our roads (which is due in part because of both previous literature and media coverage of accidents involving older drivers (Langford et al. 2008)), even though are more likely to hold “pro-traffic safety” stances on a variety of issues, including more government intervention and funding with regard to traffic safety and support for police enforcement of traffic laws compared to younger individuals (Girasek 2013).

The majority of previous studies have treated drivers aged 65 and older as a single group. However, “older senior drivers” (e.g., ages 80 and older) might differ in important ways from “younger senior drivers.” For example, both fragility (as defined by risk of death when involved in a crash) and the rate of crash involvement steadily increase with age among drivers in this age group (Li et al. 2003). In these respects, drivers over the age of 75 are substantially different from those aged 65-69 or those aged 70-74. It is of interest to know whether their attitutdes and behaviors towards driving also differ.

The purpose of the research reported here was to examine the extent and nature of the variability in driving behaviors and safety-related attitudes among drivers ages 65–69, 70–74, and 75 and older, using data from the AAA Foundation’s annual Traffic Safety Culture Index survey.

## Methods

### Data

The data analyzed for this study were from the AAA Foundation’s annual Traffic Safety Culture Index survey. The survey comprises questions about Americans’ attitudes about traffic safety issues, social norms, and driving-related behaviors. Each year, it is administered online to a sample of U.S. residents aged 16 years and older who were enrolled in GfK’s KnowledgePanel®, a nationally-representative sample of members of U.S. households recruited by standard probability-based survey methods (address-based mail and random-digit dial telephone sampling). If a recruited household lacks Internet access, GfK provides an Internet connection and hardware at no cost to the household (AAA Foundation for Traffic Safety, 2014b).

The current study analyzed data from the 2011, 2012, and 2013 Traffic Safety Culture Index surveys, administered June 6, 2011 to June 28, 2011, September 7, 2012 to September 24, 2012, and September 18 through October 3, 2013, respectively. Data were weighted to account for individual respondents’ probability being recruited into KnowledgePanel®, respondents’ probability of being selected for these specific surveys, and non-response at both stages. Post-stratification weights aligned the demographics of the sample to that of the U.S. population with respect to age, sex, race and ethnicity, education, census region, urban versus rural residence, household size, and household income. The sum of the weights added to the total survey sample size.

Respondents were asked questions related to the extent that they believed specific driving behaviors of other drivers were a threat to the respondent’s personal safety. Response categories were *very serious threat*, *somewhat serious threat*, *minor threat*, and *not a threat*; these were dichotomized as *very or somewhat serious threat* versus *minor threat or not a threat*.

To assess social norms, respondents were asked whether they considered it to be *completely acceptable*, *somewhat acceptable*, *somewhat unacceptable*, or *completely unacceptable* for a driver to perform a variety of specific behaviors; these were dichotomized as *approved* versus *disapproved*.

In questions related to support for a number of specific traffic safety interventions, respondents were asked whether they would *strongly support*, *somewhat support*, *somewhat oppose*, or *strongly oppose* each one; these were dichotomized as *support* versus *oppose*.

Respondents who reported having driven in the past 30 days were asked how often they had engaged in several specific behaviors; response options were *regularly*, *fairly often*, *rarely*, *just once*, and *never*. These data were treated as ordinal and were not dichotomized or otherwise recoded.

Number of moving violations over the last two years was coded into the following categories: *2 or more*, *1*, and *2*. For driver crash involvement, the categories were: *3 or more*, *2*, *1*, and *0*. This was done to ensure adequate numbers of observations in all categories, as very few respondents reported more than two violations or more than three crashes. One observation, in which a respondent reported having zero moving violations but nine crashes in the past two years (18 standard deviations from the mean number of crashses in the full sample), was suspected to be a coding error and was recoded as missing.

Respondents were coded as living in a metropolitan or non-metro area according to the United States Office of Management and Budget classification of the ZIP code of the respondent’s home address.

### Statistical Analysis

Respondents were categorized into three age groups: 65–69, 70–74, and 75 and older. For demographic characteristics other than age (Table 1), prevalence of crashes, violations, and injuries (Table 2), attitudes towards driving behaviors (Table 3), support for interventions (Table 4), and engagement in behaviors (Table 5), the frequency of responses was calculated. Chi-squared tests were used to assess whether the distributions of these variables varied significantly by age. The effect of age on attitudes towards driving behaviors and support for driving safety interventions were addressed using logistic regression, which yielded odds ratios.The significance of differences in the frequencies of behaviors were assessed using ordinal logistic regression.

**Table 1:** **Demographics of Survey Respondents (n = 1793), United States, 2011-2013**

	Age (years)					
	Unweighted n (weighted %)^‡^					
	65-69		70-74		≥75	
**Sex**						
Male	340	(46)	215	(48)	326	(44)
Female	388	(54)	202	(52)	322	(56)
**Race/Ethnicity**						
White, non-Hispanic	582	(72)	351	(82)	560	(85)
Black, non-Hispanic	55	(11)	21	(7)	29	(6)
Hispanic	64	(13)	21	(6)	34	(6)
2 or more races, non-Hispanic	14	(1)	12	(2)	15	(2)
Other, non-Hispanic	13	(4)	12	(4)	10	(2)
**Education**						
Less than high school	60	(12)	47	(15)	77	(16)
High school	237	(35)	156	(35)	214	(40)
Some college	148	(20)	74	(17)	122	(15)
Associate or bachelor	162	(19)	91	(20)	139	(18)
Graduate	90	(10)	34	(9)	57	(7)
Professional	31	(4)	15	(5)	39	(5)
**Marital Status**						
Married/living with partner	509	(65)	305	(75)	372	(55)
Divorced/widowed/separated	182	(30)	98	(21)	256	(42)
Never married	37	(5)	14	(4)	20	(3)
**Employment**						
Employed	159	(22)	57	(15)	50	(6)
Laid off/seeking employment	12	(2)	8	(2)	3	(1)
Retired	503	(68)	341	(82)	577	(89)
Disabled	35	(5)	8	(1)	10	(2)
Other (not working)	19	(4)	3	(1)	8	(2)
**Household Income ($)**						
<30,000	145	(24)	99	(23)	175	(28)
30,000-49,999	153	(22)	90	(21)	167	(22)
50,000-74,999	157	(21)	105	(20)	131	(17)
75,000-99,999	98	(12)	43	(12)	68	(12)
≥100,000	175	(22)	80	(25)	107	(21)
**Driven in last 30 days**						
Yes	669	(89)	391	(93)	571	(87)
No	58	(11)	26	(7)	77	(13)
**Metro Status§**						
Metro	612	(83)	365	(83)	559	(83)
Non-Metro	116	(17)	52	(17)	89	(17)
**Primary Vehicle Type**						
Car	437	(64)	259	(67)	416	(72)
Van/Mini-Van	53	(6)	37	(9)	70	(11)
SUV	118	(17)	60	(14)	69	(10)
Truck	78	(12)	43	(9)	32	(6)
Motorcycle	3	(1)	1	(1)	2	(1)

^‡^May not add to 100 % due to rounding

^§^Defined by the US Office of Management and Budget as a geographic entity with a core urban area, population >50,000.

**Table 2:** **Crashes, violations, and injuries among survey respondents aged 65 and older, United States, 2011–2013 (n = 1793)**

	Age in years		
	(weighted %)		
	65-69	70-74	≥75
Moving violations in last two years*			
0	88.2	92.8	92.6
1	10.4	6.7	7.0
2	1.4	0.6	0.2
≥3	0.1	0.0	0.3
**Crashes involved in last two years**			
0	91.9	92.8	88.7
1	7.0	6.7	9.9
≥2	1.1	0.5	1.4
**Serious injury in an MVC** ^**†**^	16.0	11.2	13.2

*P ≤ .05

^†^Over respondent’s lifespan

**Table 3:** Proportion of respondents age 65 and older who rated driving behaviors shown as unacceptable (n = 1793), United States, 2011-2013

	Age in years			
	(weighted %)			
	65-69	70-74	≥75	χ^2^ p-value
**Speeding**				
Residential driving ≥10 mph over limit	93.9	97.6	94.9	0.03
Urban driving ≥10 mph over limit^†^	88.9	95.2	93.5	0.02
School zone driving ≥10 mph over limit^†^	97.5	98.7	99.7	0.01
Freeway driving ≥15 mph over limit	85.6	89.8	86.8	0.24
**Phone-Related Behaviors**				
Talking on hand-held phones while driving	83.7	90.9	89.3	0.01
Talking on hands-free phones while driving	55.9	54.2	62.4	0.08
Texting/e-mailing while driving	99.0	99.3	98.6	0.55
Checking social media while driving	98.6	99.2	99.2	0.68
**Impaired Driving Behaviors**				
Driving after drinking enough to be impaired	99.0	99.6	99.4	0.64
Driving within an hour of using cannabis^‡^	96.6	99.1	97.7	0.46
Driving after using both cannabis and alcohol^‡^	98.3	100.0	99.6	0.80
Driving while drowsy	99.1	98.9	99.1	0.96
**Miscellaneous Behaviors**				
Not wearing a seatbelt	90.7	96.5	92.8	0.04
Driving through a light that just became a red	96.8	99.0	97.2	0.03

^†^Question not asked in 2011 survey. For these, n = 1275

^‡^Question asked in 2013 only. For these, n = 451

**Table 4:** Support for driving interventions among respondents age 65 and older, United States, 2011–2013 n = (1793)

	Age in years			
	(weighted %)			
	65-69	70-74	≥75	χ^2^ p-value
**Speed Cameras Ticketing: Driving ≥10 mph over the speed limit in** ^**†‡**^				
Residential areas	55.8	58.3	70.7	0.02
Urban areas	50.7	50.4	67.2	0.01
School zones	67.1	71.3	82.2	0.01
Freeways	47.9	46.6	59.7	0.08
**Phone-Related Laws**				
Law against texting/e-mailing while driving	95.3	92.9	97.6	0.01
Law against using hand-held cell phone while driving	83.8	83.2	89.1	0.07
Law against any phone use while driving	65.6	65.4	72.2	0.08
**DWI-related laws**				
Lower legal BAC limit from .08 to .05	60.1	66.7	59.0	0.58
Requiring new cars to have technology that prevent them from starting when driver is above legal BAC limit	76.6	77.7	86.5	0.01
Ignition locks for DWI offenders, even after the 1^st^ time	87.6	88.2	89.1	0.69
**Red Light Cameras** ^**†‡**^				
In residential areas	63.6	63.5	84.0	<0.01
In urban areas	66.3	70.1	79.3	0.06
**License Renewal Laws for Senior Drivers** ^**‡**^				
In person license renewal at age 75 & older	74.2	72.3	78.6	0.25
Health screening to renew license at age 75 & older	76.8	71.4	77.0	0.37
**Other Laws**				
Motorcycle helmet laws	85.8	91.4	95.1	<0.01
Requiring states to publish annual maps with locations of crashes	57.0	59.2	58.0	0.86
Regulations on in-car technology^‡^	55.1	50.4	60.7	0.10

^†^All respondents answered only 3 of 6 randomly selected questions related to support for camera laws

^‡^Question not asked in 2011 survey

**Table 5:** **Engagement in unsafe driving behaviors among respondents age 65 and older who drove in last 30 days (n = 1631), United States, 2011-2013**

	Age in years			
	(weighted %)			
	65-69	70-74	≥75	χ^2^ p-value
**Sped by 15 mph or more on a freeway**				
Fairly Often/Regularly	7.7	6.5	8.4	
Just Once/Rarely	34.1	35.0	35.8	
Never	58.2	58.5	55.8	0.69
**Sped by 10 mph or more in a residential area**				
Fairly Often/Regularly	6.0	7.7	7.8	
Just Once/Rarely	34.7	33.8	40.3	
Never	59.4	58.5	51.9	0.10
**Read text/e-mail while driving**				
Fairly Often/Regularly	0.8	0.8	0.8	
Just Once/Rarely	8.1	5.1	2.4	
Never	91.1	94.1	96.7	0.007
**Typed text/e-mail while driving**				
Fairly Often/Regularly	0.4	0.3	0.6	
Just Once/Rarely	3.3	1.0	0.9	
Never	96.3	98.7	98.5	0.02
**Drove without a seatbelt**				
Fairly Often /Regularly	5.7	6.1	5.1	
Just Once/Rarely	12.7	9.7	19.9	
Never	81.6	84.3	75.1	0.04
**Drove while drowsy**				
Fairly Often/Regularly	1.7	0.7	0.9	
Just Once/Rarely	20.2	22.3	27.6	
Never	78.1	77.0	71.6	0.12
**Drove through a red light**				
Fairly Often/Regularly	1.1	1.0	0.6	
Just Once/Rarely	29.9	29.9	37.0	
Never	69.0	69.1	62.5	0.13
**Used a cell phone while driving‡**				
Fairly Often/Regularly	12.2	12.1	6.6	
Just Once/Rarely	39.7	32.4	27.5	
Never	48.1	55.5	65.8	<.0001
**Used internet while driving§**				
Fairly Often/Regularly	0.8	1.8	0.9	
Just Once/Rarely	1.0	0.4	1.5	
Never	98.3	97.9	97.7	0.88

^‡^Refers to any cell phone use

^§^Question not asked in 2011 survey

Bivariate and multivariate logistic models were estimated to assess whether any apparently age-related differences persisted after adjusting for other demographic variables besides age, namely the ones seen in Table 1 Variables in these models were not modified from the way in which they were coded in the raw data set.

The sample was comprised of 1,793 respondents aged 65 years and older; however, the number of valid responses varied by individual survey question, as some respondents refused to answer some questions (refusals were coded as missing data), several questions were not asked in all three years of the survey, some questions were asked of a random sub-sample of respondents rather than all respondents to avoid imposing excessive respondent burden, and questions regarding recent driving behaviors were only asked of respondents who reported having driven at least once in the 30 days before they completed the survey.

All analyses were done on the weighted survey data. Analyses were performed using statistical software SAS version 9.4 (SAS Institute Inc., Cary, NC). Statistical significance was set at p ≤ .05.

## Results

The weighted sample had a mean age of 72.8 years with a standard deviation of 6.1 years and ranged from 65 years to 95 years. The sample was weighted to be representative of the population of the United States over the age of 65 with regard to sex, race/ethnicity, education level, employment status, household income, metropolitan status, and marital status. The sample’s demographic characteristics can be found in Table 1. Drivers aged 75 and older were more likely to be white, non-hispanic, and less likely to be married or employed. They were also more likely to drive a car as opposed to a van, mini-van, SUV or truck.

### Crashes and Violations

Among respondents who reported the number of moving violations and crashes over the past two years, 90.8 % reported no moving violations and 91.0 % had not been involved in a crash as a driver in the last two years (Table 2). While drivers 65–69 years old reported more moving violations than the other two age groups (p < 0.05), there was no statistically signficant difference in crash involvement between the three age groups. Along the lifespans of survey respondents, 31.8 % reported knowing a family member or close friend seriously injured in a motor vehicle crash and 13.9 % had themselves been seriously injured in a motor vehicle crash.

### Attitudes Towards Driving Behaviors

A vast majority of respondents strongly disapproved of all of the unsafe driving behaviors included in the survey. Respondents age 65–69 expressed slightly lower levels of disapproval of several of the behaviors, though, compared with respondents aged 70–74 and 75 and older.

Overwhelming majorities of drivers aged 65 and older disapprove of speeding, whether it be on freeways, in residential areas, in urban areas, or in school zones. Drivers aged 70–74 were more likely to disapprove of speeding in residential areas and in urban areas than drivers aged 65–69 or 75 and older. The difference between the 65–69 year olds and those 75 years old or more was not significant on these measures. As age category increased, disapproval of school-zone speeding increased. The difference between those 75 and older and those 65–69 was statistically significant; the difference between those 65–69 and those 70–74 was not. Although some of these differences were statistically significant, they were not different in a meaningful way as disapproval for these behaviors was virtually universal across all three age groups.

The proportion of older Americans disapproving of texting and e-mailing while driving and checking or posting on social media while driving were nearly 100 % and did not vary between the groups. However, while ranging from approximately 84 % to 91 % disapproval, respondents 70–74 years old and respondents 75 and older were significantly more likely than drivers ages 65–69 to disapprove of drivers talking on hand-held phones. Although the rate of disapproval of talking on hands-free phones while driving was noticeably higher among the oldest group (62 %) than the younger two (56 %, 54 %), the differences were not statistically significant (p = .08). Impaired driving behaviors (driving when one may have had too much to drink, driving one hour after using cannabis, and driving after using both cannabis and alcohol) and driving while drowsy garnered nearly 100 % disapproval among those in the study sample. There was no difference between the age groups with regard to their views of impaired driving.While more than 90 % of respondents in all age groups disapproved of driving without wearing a seatbelt and driving through a red lights when it would have been possible to stop safely, the rate of disapproval was highest among drivers ages 70–74 for both behaviors (Table 3).

Adjustment for other demographic characteristics besides age had little effect on the magnitude or statistical significance of the relationships between any of the above-mentioned variables and age group.

### Support for Safety Interventions

The participants were asked about their support for various traffic safety interventions such as traffic cameras, cell phone laws, DWI laws, and older driver license renewal laws (Table 4). Large differences were observed by age, especially when comparing respondents ages 75 and older to the two younger age groups.

For questions about support for speed cameras, speeding was defined as “driving more than 10 mph over the speed limit” for the respective location. Using cameras to automatically ticket drivers who speed in residential areas found majority support across all three groups, but the age 75 and older group supported using them more than 65–69 year olds and 70–74 year olds did. The same pattern was found with speed cameras in urban areas, which found majority support across all three groups, with support again strongest among the oldest respondents. The pattern also held for speed cameras in school zones, although the only difference that was significant was that between support among those 75 and older and those 65–69 years old. This suggests consistently stronger support among the 75 and older group. The proportion of respondents supporting speed camera ticketing drivers who speed on freeways was below 50 % among those between 65 and 74, but nearly 60 % among those aged 75 and older. However, the difference was not statistically significant (p = .08).

Support for laws against using cell phones while driving was strong across all age groups, but generally tended to be stronger among the oldest respondents. A law restricting the use of any and all cell phones generally received the lowest levels of support, while a law restricting only hand-held cell phone use was more popular, and a law against text messaging or emailing while driving received virtually universal support across all three age groups.

Support for lowering the legal limit for blood alcohol content (BAC) while driving in the United States from .08 g/dL to .05 g/dL was 61 % among senior respondents. While support was highest among those 70–74 years old (67 %), there was no statistically significant difference between the age groups (note that this question was only asked in 2013 and thus analysis is based on a much smaller number of responses than other items). Nearly 90 % of those surveyed supported requiring alcohol-ignition locks for all drivers convicted of driving while intoxicated, including first-time offenders, and this did not vary by age. This support was uniform across the age categories. On the other hand, support for requiring alcohol ignition interlock technology in all new cars did vary by age group, with support being much higher among respondents ages 75 and older than among younger respondents.

Although not quite reaching conventional levels of statistical significance, there was a clear pattern of support for using cameras to automatically ticket drivers who run red lights in urban areas, as 66 % of respondents ages 65–69, 70 % of those ages 70–74, and 75 % of those ages 75 and older expressed support. Support for using red light cameras on residential streets was similar among drivers ages 65–69 and 70–74, but much higher among drivers ages 75 and older.

Laws requiring drivers over 75 to renew their license in person and requiring they pass a medical screen to remain licensed received support from over 70 % of respondents across all age groups. In an interesting trend, support for these measures are at their lowest point right before the age specified by these laws (ages 70–74) and at their highest point after (age 75 and older). However, these differences did not reach statistical significance.

Support for laws requiring states to publish maps annually with locations of motor vehicle crashes and for federal government regulations on in-car technology was consistent across age groups. Support for motorcycle helmet laws increased with age: 86 % of drivers ages 65–69, 91 % of drivers ages 70–74, and 95 % of drivers ages 75 and older expressed support for laws requiring all motorcyclists to wear a helmet.

Despite not reaching statistical significance in simple bivariate comparisons, differences in support for using speed cameras on freeways and using red light cameras in urban areas did vary significantly by age after adjustment for other demographic characteristics besides age. Adjustment for other demographic characteristics did not change the statistical significance of any of the other above-mentioned variables.

### Engagement in Unsafe Behaviors

Self-reported internet use and reading and sending of text messages while driving among drivers older than 65 was extremely rare. Drivers aged 75 and more reported significantly lower levels of reading text messages while driving than did the other age groups, and drivers ages 70–74 and 75 and older both reported significantly lower levels of typing text messages while driving than did drivers ages 65–69. Internet use while driving was virtually non-existent in all three age groups and did not vary significantly by age. Talking on cell phones while driving was much more common: more than half of all drivers ages 65–69 reported having talked on a cell phone while driving at least once in the past 30 days, including 12 % who reported having done so fairly often or regularly. Cell phone use while driving was slightly less common among drivers ages 70–74 and significantly less common among drivers age 75and older.

Self-reported speeding was relatively common among all three age groups. On freeways, 42-46 % reporting having driven 15 or more mph over the speed limit at least once in the past 30 days and 7-8 % reporting having done so fairly often or regularly, which did not vary significantly by age. Similarly, 41-48 % reported having exceeded the speed limit by 10 or more mph on a residential street at least once in the past 30 days, with 6-8 % reporting having done so fairly often or regularly. While 31-38 % of drivers reported having driven through a red light when they could have stopped safely at least once in the past 30 days, very few (1 %) reported having done this fairly often or regularly. While variation by age was not statistically significant for any of these variables, it was interesting and surprising to note that drivers ages 75 or older were more likely than drivers in either of the younger age groups to report speeding on residential streets and running red lights.

Most older drivers reported always wearing seatbelt when they drove, however, 18 % of drivers ages 65–69, 16 % of those ages 70–74, and 25 % of drivers ages 75 and older reported having driven without wearing a seatbelt at least once in the past 30 days; variation by age was statistically significant, with the oldest drivers the most likely to report this behavior.

The oldest drivers were somewhat more likely to report having driven when they were so tired that they had a hard time keeping their eyes open at least once in the past 30 days (18 % reported having done so). While non-significant in the bivariate comparison, drivers ages 75 and older were significantly more likely to report drowsy driving than drivers in the two younger age groups after adjusting for other demographic characteristics.

## Discussion

The results show that older people, especially those aged 75 and older, are supportive of elements of a positive traffic safety culture. There is both strong disapproval of negative driving behaviors and strong support for interventions that would correct these behaviors. In addition, they show low levels of participation in the same behaviors that they express disapproval of. It is particularly noteworthy that more than 7 in 10 seniors support both mandating in-person license renewals and medical screenings for those drivers over the age of 75, a figure boosted by respondents over age 75. Although one could argue that seniors might overestimate their ability to pass a medical screening, the fact that so many are willing to accept more stringent driver license renewal requirements suggests that traffic safety is an important value among this group.

The results indicate that while the youngest of the older population examined here—those ages 65-69—are quite similar to those ages 70–74 and 75 and older with respect to their attitudes, opinions, and self-reported driving behaviors, there are also some key differences that may have important implications for traffic safety research and programs. In particular, the results highlight a difference in opinion between the youngest group of older drivers and the oldest group regarding speeding. The youngest group is more likely to speed and find it an acceptable behavior, and they are much less likely to support speed cameras. They also showed very different views on issues related to cell phone use. Cell phone use in the last thirty days was significantly more common among the youngest group of older adults (aged 65–69), and they were significantly more likely to read and type text messges.This may be a consequence of being generally more familiar and comfortable using cell phones, or related to comfort with multi-tasking while driving. Either way, it is a trend that should be moniotred as this group of Americans continue to age.

The findings about those age 70–74 (the “middle” group) may be most interesting. In some ways, they closely mirrored those aged 65–69. For example, their support for red light and speed cameras and proclivity to use a cell phone or drive drowsy were nearly identical. But in some ways (the prevalence of moving violations, their opinion on hand-held cell phone use while driving), they were more like those 75 or older. If the results are indeed the result of an age effect, then the ages of 70–74 may be the time when a shift in opinions occurs. As evidenced in Table 1, this is a time when the sample begins to contain a much higher percentage of retired individuals. However, adjusting for employment status did not make a significant impact on the observed results.

Since the relationship between age and the outcomes analyzed remained when adjusted for other demographic characteristics including sex, race, education, marital status, income, job status, type of car driven, and metropolitan status, there is a higher degree of confidence that age is an important factor in these relationships. However, there were other demographic factors that were independently related to the outcomes, which could be a topic for future work. For example, differences were observed in the responses of men versus women to many of the questions about attitudes and perceptions towards both driving behaviors and interventions. Sex is a particularly relevant demographic characteristic among older people because of wide disparities that have been observed in driving cessation between men and women (Choi et al. 2013). However, analysis adjusting for sex only showed that it was not a confounder of the relationships found between age and the outcomes in this study.

There were many topics on which survey responses were only weakly related or completely unrelated to age among the population aged 65. The absence of significant differences between the age groups for some items should not be interpreted as evidence that older drivers should all be treated as one collective group. Despite many physiological and demographic similarities between the 65–69 year olds, 70–74 year olds, and those 75 and older, it is evident that attitudes and behaviors differ with respect to age on many dimensions that are important to improving traffic safety.

Despite their concern for safety, many seniors were willing to engage in behaviors they found unacceptable. For example, while more than 95 % of all respondents said speeding in excess of 10 mph over the speed limit on residential streets was unacceptable, more than 40 % reported having done so in the past 30 days, including 6-8 % who reported doing this fairly often or regularly. And despite of the fact that nearly all respondents found red light running unacceptable, 1 in 3 respondents reported that they had run a red light in the past month on an occasion when they admitted that they would have been able to stop safely. This shows that while our results may show one age group to have a more positive attittude towards safety than another on a given issue, that does not make them safer. It is possible that the rise of advanced driver assistance systems technologies that warn drivers about issues such as lane deviation and being too close to another car will help prevent senior drivers from being involved in crashes. Previous research has found that a person giving driving directions and help to older drivers reduces the rate at which they commit errors, which the researchers noted suggests efficacy for navigation systems in the cars of older drivers (Wood et al. 2009).

These findings have implications for those who want to make driving safer. They show that older Americans, particularly ones over age 75, are important allies in their mission. That of course assumes that age leads to a change in attitudes. If those drivers aged 65–69 do not change with age and are representative of Baby Boomers, the older drivers of ten to twenty years from now may differ in important ways from those on the road today. That being said, this analysis demonstrates that there are some unsafe driving behaviors that still have a high level of acceptance, even among seniors, which suggests seniors may underestimate the risk associated with these behaviors.

### Limitations and Future Work

The large size of the sample of senior drivers analyzed for the current study made it possible to detect even small differences between age groups; however, this resulted in even some trivially small differences being statistically significant at conventional levels (i.e., p < .05). For example, 97.5 % of respondents aged 65–69, 98.7 % of respondents aged 70–74, and 99.7 % of respondents aged 75 and older reported that they consider it unacceptable to drive 10 mph over the speed limit in a school zone. While these differences were statistically significant (P = .01), owing primarily to the large sample size, there clearly are not important age-related differences among seniors in attitudes about speeding in school zones: virtually all of them rate this as unacceptable.

Conversely, there were some unexpected results that, while not statistically significant, may warrant at least some follow-up. For example, the finding that drivers ages 75 and older were more likely to report running red lights than respondents in either of the other two age groups (38 % vs. 31 %) was unexpected. This was not stastically significant (p = .13) and may simply be due to random sampling variability, or that the oldest drivers were simply more honest in reporting their own behavior (i.e., that the younger seniors did this even more but did not want to admit it). However, if this pattern of older drivers being more likely to run red lights is observed in future studies as well, it may warrant increased attention. Although not believed to be related to red-light running, previous research has shown that crash rates of older drivers begin to increase at around age 75 (Tefft et al. 1995).

A key limitation is the fact that because this is a survey, all answers are self-reported. Respondents may be uncomfortable expressing approval of or admitting engagement in unsafe driving behaviors. They may also be hesitant to express opposition to traffic regulations. For example, studies have constitently found self-reported seatbelt use to exceed actual seatbelt use (Nelson 1996, Zambon et al. 2008). This is likely the result of social desireability bias, or a bias to report what is perceieved to be “correct” or socially acceptable (Fisher, 1993). However, recall bias is a potential issue as well. This poses a potential issue should this bias be differential according to age cohort, which would have impacted the results. Such impact of social desirability bias may be especially true because respondents were asked about how they believe others feel about negative driving behaviors before they were asked how they themselves felt. In fact, how respondents thought “most other people” felt regarding driving behaviors and their own opinions about those same behaviors were often strongly correlated.

In addition to assessing personal opinions seperately from assessing perceptions of norms, future work may want to focus on further stratification of those aged 65 and above, particularly among the group aged 75 and older. This group contains a range of ages upwards of 25 years and is a time of great change, particularly in the rate of licensure and number of miles driven per year. Such stratification was not practical in the current study due to a limited number of survey respondents older than 80 and very few older than 85, which would have yielded insufficient power to asess the outcomes of interest. A key question is whether these results are the consequence of an age effect or a cohort effect. In other words, we cannot say whether people change with age, or whether different generations have different viewpoints, which are manifesting in the results. For example, this study can not answer the question of whether the current 65–69 year olds start to use their cell phones less as they age and mirror their older peers in five years or if cell phone use among older drivers will become more ubiqitous in the future. After all, many of the 65–69 year olds in this sample are Baby Boomers, while none of those 75 and older are. This is a potential topic for future work.

## Conclusions

In conclusion, the results show that seniors have a pro-traffic safety culture as a group. There appear to be some key differences between younger seniors (i.e.-those age 65–69) and older seniors (those over 75), however, they generally seem to hold very similar views on traffic safety. Noteably, seniors strongly support laws and regulations that would ostensibly make roads safer, even ones that would directly affect them (i.e. requiring medical screenings and in-person license renewals among older drivers). These are important findings at a time when the proportion of our population over the age of 65 is quickly expanding and 65 year olds are becoming healthier and more active than ever.

Despite of these positive attitudes and beliefs, they still do not completely translate into positive behaviors. This was evident in the large proportion of respondents who said they disapproved of unsafe driving behaviors that they reported engaging in themselves.
